# Editorial: Neuromodulation in basic, translational and clinical research in psychiatry, volume II

**DOI:** 10.3389/fnhum.2023.1212625

**Published:** 2023-05-19

**Authors:** Kay Jann, Ryouhei Ishii, Shun Takahashi, Sunichiro Ikeda

**Affiliations:** ^1^USC Stevens Neuroimaging and Informatics Institute, Keck School of Medicine of USC, University of Southern California, Los Angeles, CA, United States; ^2^Department of Occupational Therapy, Graduate School of Rehabilitation Science, Osaka Metropolitan University, Habikino, Japan; ^3^Department of Psychiatry, Graduate School of Medicine, Osaka University, Suita, Japan; ^4^Department of Neuropsychiatry, Kansai Medical University, Osaka, Japan

**Keywords:** magnetoencephalography (MEG), transcranial magnetic stimulation (TMS), electroconvulsive therapy (ECT), major depression (MD), anesthesia, epigenetics, neuromodulation

The term “neuromodulation” is a wide-ranging concept that could be technically applied to any medical, surgical or physiological therapy designed to modulate the nervous system's function. In clinical neuroscience, however, the concept of neuromodulation is specifically used to refer to therapies that employ the targeted administration of an electric current or magnetic field. These techniques include electroconvulsive therapy (ECT), vagus nerve stimulation (VNS) and repetitive transcranial magnetic stimulation (rTMS). More recently, research has been conducted using transcranial direct current stimulation (tDCS), trigeminal nerve stimulation (TNS) and deep brain stimulation (DBS). In order to develop more promising therapies for psychiatric disorders, translational research bridging basic and clinical sciences should be pursued.

The aim of this Research Topic is to provide a forum for researchers interested in basic, translational and clinical research on neuromodulation in psychiatric disorders, to provide an inclusive perspective on neuromodulation, and to encourage discussion and collaboration across research areas. Four manuscripts (three original papers, one mini-review) have been accepted for this theme, all presenting significant new findings and research methods, such as the effect of inhalational anesthesia on ECT, a systematic review of epigenetic mechanisms associated with ECT, prediction of treatment response to rTMS in depression, and magnetoencephalography (MEG) analysis during automatic lexical processing of Japanese kanji compound words. The objective of each investigator is to elucidate the mechanisms underlying the pathogenesis of psychiatric disorders using basic research techniques and to apply the discoveries to the care of actual patients.

Electroconvulsive therapy (ECT), which delivers a short burst of electrical impulses to the brain under anesthesia, is the most effective therapeutic intervention available for the treatment of medication-refractory major depressive disorder (MDD), bipolar disorder (BD), suicidal behavior, severe agitation, catatonia, and clozapine-refractory schizophrenia. The effectiveness of ECT is three times greater on average than that of drug therapy, providing up to 60% improvement in symptoms in patients with MDD, and response to therapeutic intervention is usually more rapid than with current medications, however, the mechanism of therapeutic action has not been clearly understood.

While anesthetics, such as sevoflurane and thiopental, play an important role in ECT, the seizure adequacy and clinical efficacy of anesthetics on ECT are still unclear. Yatomi et al. tried to examine the seizure tolerance and clinical effectiveness of sevoflurane compared with thiopental during ECT in people with mood disorders (MD). They conducted a single-center study with a retrospective chart review. People with MD who had received ECT and administered sevoflurane (*n* = 26) or thiopental (*n* = 26) were enrolled. ECT outcomes and factors were assessed and compared between the two groups using propensity score (PS) matching. They found that people with MD who received sevoflurane required significantly more stimulations and more sessions and had more ineffective seizures than those who received thiopental. Both groups had similar remission and response rates. They therefore suggested that sevoflurane should be used with more caution in ECT and only when the clinical indications are well established.

Although the mechanism of ECT is not well understood and the number of studies is relatively poor, the development of biomarkers for response to ECT is a very attractive area in epigenetics. Castro et al. performed a review of previous studies published in the medical and scientific literatures that investigated the epigenetics of ECT in samples obtained from peripheral blood of people suffering from MDD. A systematic review was conducted according to the PRISMA guidelines. They enrolled nine studies and found that seven studies investigated DNA methylation and three studies assessed microRNAs (miR). Overall, most studies were explorative, with small sample sizes and high variability in study design, especially about ECT protocols, molecular biology methods, and epigenetic results. The candidate genes investigated with some evidence of an associated effect on treatment response to ECT were, TNKS, S100A10, FKBP5, RNF213M, miR-106a, miR-24, miR-126 and BDNF. They concluded that these findings supported previous studies, suggesting that epigenetic pathways may be important in the molecular mechanisms underlying the mechanism of ECT.

TMS is a non-invasive brain stimulation technique that generates a high- or low-intensity magnetic field that is supposed to regulate brain cortical excitability. rTMS applies repetitive TMS pulses to a targeted area of the brain. rTMS has been investigated as a potential therapeutic modality for several neurological and psychiatric disorders. The neuromodulation effects are dependent on various parameters of rTMS, such as stimulation frequency, stimulation intensity, stimulation duration, cortical target, number of trials, and individual patient characteristics, such as age, disease severity, medical history, and symptoms. Generally, rTMS has been categorized as high frequency (>1 Hz), which enhances cortical excitability, and low frequency (<1 Hz), which suppresses cortical excitability.

Cognitive impairment is common in people with both unipolar and bipolar depressive disorders (UDD and BDD, respectively). As assessment of cognitive function becomes more widely available to both researchers and practitioners, it may be useful to target these symptoms in treatment and to use them at treatment entry as potential markers of treatment response. In a retrospective observational and naturalistic study, Rostomi et al. evaluated their own data from 120 people with UDD (*n* = 56) and BDD (*n* = 64) who were treated with 20 sessions of bilateral rTMS, consisted of 10 Hz over the LDLPFC and 1 Hz over the RDLPFC. They evaluated depressive symptoms, sustained attention, working memory and executive function at baseline and at the completion of the rTMS treatment course. They found that more than half of all these subjects (*n* = 64), especially 53.1% of UDD subjects (*n* = 34) and 46.9% of BDD subjects (*n* = 30), showed treatment response. They also reported that bilateral rTMS significantly improved several cognitive functions (attention, working memory and executive function) excluding visual memory and led to more changes in working memory in UDD subjects compared to BDD subjects. More marked improvements in working memory were found in patients who responded, and visual memory, age and sex were determined to be predictors of treatment response. Working memory, visual memory and age were identified as predictors of response to treatment in BDD and UDD subjects, respectively. They concluded that bilateral rTMS significantly improved several cognitive functions and depressive symptoms in UDD and BDD patients, probably by modifying top-down cognitive control mechanisms and by processing negative emotional biases.

When the semantic dimension of a word is processed automatically during lexical processing, the response to a preattentive presentation of a semantically incorrect word variant may show greater brain activity than the response to the semantically accurate word presented. The Japanese kanji compound, which consists of two or more characters, is suitable for examining this problem. The written Japanese language is composed of kana sounds, which are similar to the alphabet, and ideograms, known as kanji, which are more typically processed by whole-word reading. These ideograms often show up in the form of a kanji compound. A kanji compound consists of a specific kanji combination, and an invalid combination is considered non-lexical as a kanji compound, even though the single kanji character could be lexical. Kanji compounds, however, generally express not only concepts but also their associated meanings. Therefore, this feature of kanji compounds can be used as an experimental setting for semantically incorrect variation. It is important to note that since Kanji reveals reading and/or writing impairments in Japanese, understanding the visual recognition of Kanji helps to understand the underlying mechanisms of fluent reading capability. For these reasons, they focused on the semantic processing of visual kanji compounds.

To investigate this phenomenon for Japanese kanji compounds, where lexicality is associated with semantic connotations, Egashira et al. used three types of deviants: font differences, lexically correct or incorrect Japanese kanji compound words, and pseudo-kanji characters that were modified from correct and incorrect compounds. They applied MEG to elucidate the spatio-temporal patterns of the relevant brain areas. The three types of stimuli listed above, with 20% deviations, were displayed during the MEG recordings. They found clear occipital pole activity during the recognition of font type variations within 250 ms of stimulus onset. The reported that no significant activity was observed during the detection of lexically correct or incorrect Kanji compounds or pseudo-Kanji character deviations, although activity in the posterior transverse region of the collateral sulcus (pCoS)—a fusiform adjacent area—was larger for the recognition of lexically correct Kanji compounds than for the recognition of pseudo-Kanji characters. They concluded that the automatic recognition of deviance in kanji compounds may be restricted to a lower-level characteristic, such as the thickness of the stimulus line.

The Research Theme, in summary, provided a broad overview of the clinical applicability of the most recent research discoveries to psychiatric disorders and cognitive function, and offered an insight into the translation of basic research into current psychiatric clinical practice. As a result, these papers illuminated the pathogenesis of psychiatric disorders by using basic research techniques and by applying the novel findings to the treatment of real patients, and suggested new directions for exploring the new frontiers of basic and clinical neuroscience for clinical application in psychiatry.

## Author contributions

KJ and RI discussed about this Research Topic and wrote the manuscript. ST and SI gave significant advice and helped the editing the manuscript.

